# Interaction of interregional O_3_ pollution using complex network analysis

**DOI:** 10.7717/peerj.12095

**Published:** 2021-09-07

**Authors:** Qiang Zhang, Yunan Zhu, Dianxiang Xu, Jiaqiong Yuan, Zhihe Wang, Yong Li, Xueyan Liu

**Affiliations:** 1 Computer Science and Engineering, The Northwest Normal University, Lanzhou, Gansu, China; 2 Mathematics and Statistics, The Northwest Normal University, Lanzhou, Gansu, China

**Keywords:** Air quality, Complex network, Time series, Node importance, Transfer entropy, Precise governance

## Abstract

In order to improve the accuracy of air pollution management and promote the efficiency of coordinated inter-regional prevention and control, this study analyzes the interaction of O_3_ in Qilihe District, Lanzhou City, China. Data used for analysis was obtained from 63 air quality monitoring stations between November 2017 and October 2018. This paper uses complex network theory to describe the network structure characteristics of O_3_ pollution spatial correlation. On this basis, the node importance method is used to mine the sub-network with the highest spatial correlation in the O_3_ network, and use transfer entropy theory to analyse the interaction of pollutants between regions. The results show that the O_3_ area of Qilihe District, Lanzhou City can be divided into three parts: the urban street community type areas in urban areas, the township and village type areas in mountain areas and the scattered areas represented by isolated nodes. An analysis of the mutual influence of O_3_ between each area revealed that the impact of O_3_ on each monitoring station in adjacent areas will vary considerably. Therefore these areas cannot be governed as a whole, and the traditional extensive management measures based on administrative divisions cannot be used to replace all other regional governance measures. There is the need to develop a joint prevention and control mechanism tailored to local conditions in order to improve the accuracy and efficiency of O_3_ governance.

## Introduction

The sustainable development of the environment is closely related to the development of the city. Furthermore, a sustainable and healthy environment can provide good development conditions for the city and its residents. However, with the acceleration of the urbanization process, the rapid development of the secondary industry, and the rapid increase in the number of motor vehicles, the degree of urban environmental pollution has also increased. As a result, the occurrence of severely polluted weather in some areas is frequent. Photochemical smog pollution is considered as one of the negative impacts of the rapid urbanization which occurred some years ago, especially in China. Atmospheric pollution through smog formation adversely affects the life and health of urban residents. To ensure sustainable urban development, there is the need to address the problem of environmental pollution “urban diseases”. Research shows that while the concentration of PM pollutants (PM_2.5_ and PM_10_) in China has decreased consistently, the concentration of O_3_ has taken an upward trend. For instance, the concentrations of PM_2.5_ and PM_10_ in China decreased by 6.5% and 5.1% year-on-year respectively in 2017 whereas O_3_ concentrations increased by 8% year-on-year. The forms of pollution are regional and Continuous. To improve the efficiency of environmental governance, China has been strengthening regional joint prevention and control efforts in recent years. However, according to the ACAC (2019), the proportion of cities that meet the current air quality standards in China has shown a downward trend year by year. The report revealed that cities that met O_3_ standards had fallen from 67.8% in 2018 to 65.4% in 2019. Furthermore, the annual average concentration of O_3_ and the number of days exceeding the standard keep rising. The report also showed that O_3_ levels increased from 149 µg/m^3^ (33.4%) in 2017 to 151 µg/m^3^ (43.5%) in 2018 indicating that there is an urgent need to address O_3_ pollution in China. With the continuous strengthening of joint prevention and control measures, air pollution has not achieved the expected results, which is still a daunting challenge for environmental managers. Since O_3_ formation is caused by the photochemical reaction of CO, NO_x_, and volatile organic compounds (VOCs) under ultraviolet irradiation, it can be assumed that any change of PM_2.5_ concentration might affect the change of O_3_ concentration to a great extent. Thus, O_3_ can be considered as having both regional interactions and spatial characteristics. At present, a large number of studies have focused on mutual pollution in urban agglomerations or across the country. In fact, pollution generally originates in a certain local area. In other words, only by identifying the key areas of air pollution in the region, can we achieve low-cost, rapid, and precise governance and ensure the effectiveness of the joint prevention and control mechanism. However, limited by data samples and research methods, existing research rarely reveals the interaction mechanism of air pollution in a certain local area (small spatial scale), resulting in related governance methods that are based on blind governance based on administrative divisions. Therefore, research on the interaction of O_3_ pollution in urban areas is critical to effectively manage O_3_ pollution in urban areas.

## Literature Review

### Temporal and spatial analysis of air pollutants

Many research shows that atmospheric pollution has spatial mobility and regional characteristics (Li et al., 2017; Ren et al., 2010; Zhang et al., 2014). Gao et al. (2016) used an online coupled weather research and predictive chemistry (WRF-Chem) model to simulate a haze weather process that occurred in the North China Plain (NCP) in January 2010. The outcome of the study showed that the hazewere mainly due to NCP air pollutants from heavy emissions and stable weather conditions in winter. Results also revealed that other cities contributed 64.5% of Beijing’s PM_2.5_, most of which came from southern Hebei, Tianjin, Shandong, and Henan. Wang et al. (2015) have developed a software SMAT-CE that combines observation data with model data to evaluate air quality through spatial statistical methods, aiming to provide better scientific support for air quality management. On the other hand, Yan et al. (2019) analyzed the air pollutants in Shanghai, China, they found PM_2.5_ and O_3_ to be the main pollutants in Shanghai. In addition, the researchers observed that the severely polluted weather occurred in the first half of the year although the winter was more severe. At the same time, the author also found that the linear relationship between PM2.5 and PM10 in Shanghai and the seasonal changes. Chen et al. (2020) used the NAQPMS method to analyze the PM2.5 situation in the North China Plain in December 2017 and analyzed the pollution of the coal yard to the surrounding areas during different periods. Li et al. (2015) used the CAMx model to analyze PM2.5 emissions in the Beijing-Tianjin-Hebei region of China and found that air pollution is mainly affected by local emissions and will become serious under adverse weather conditions.

Previous studies on the diffusion and transport of air pollutants in China have mainly focused on PM_2.5_ because it was the main air pollutant in the past. However, in recent years, O_3_ has attracted a lot of attention due to its increasing levels over the years. Several studies have been conducted on O_3_ in various regions in China. Zhang et al. (2008) carried out an intensive field activity in the Pearl River Delta (PRD), namely, the “Pearl River Delta Air Quality Regional Comprehensive Test Plan (PRIDE-PRD2004)” and found that the photochemical production efficiency of O_3_ is highly non-linear. This nonlinear relationship between precursor photochemical reactions is of great significance for the formulation of O_3_ control strategies. Zhang et al. (2007) analyzed O_3_ data obtained during the air monitoring tests conducted in Hong Kong and the Pearl River Delta in the autumn of 2002. The study was conducted to clarify the relationship between ground O_3_, pollution precursors (NO_y_, CO, and VOC), and cross-border transportation. Results of the study showed that the production of O_3_ in most parts of Hong Kong was limited by VOC and high concentrations of NO can inhibit the production of O_3_. Xing et al. (2018) develop a new method by fitting multiple simulations of a chemical transport model (*i.e*., Community Multiscale Air Quality Modeling System, CMAQ) with a set of polyno-mial functions (denoted as “pf-RSM”) to quantify responses of ambient PM2.5 and O3 concentrations to changes in pre-cursor emissions. Battista et al. (2016) analyzed several pollutants in Rome in 2015 using different technologies and they found that O_3_ had a high concentration in summer, and the dependence between O_3_ and C_6_H_6_ was complex. The study also shows that the reduction of C_6_H_6_ under certain thresholds can lead to an increase in O_3_. Fang, Wang & Wang (2019) used O_3_ data and meteorological data from 2013 to 2017 in Changchun to analyze the relationship between the spatiotemporal changes of O_3_ concentration and meteorological factors. The result showed that the southern Changchun may be affected by the O_3_ impact of long-distance transport of pollution. Yan et al. (2016) based on the WRF-CMAQ air quality model system, combined with the air pollutant emission inventory in the Yangtze River Delta region, they constructed a response surface modeling (RSM) between O_3_ and its precursors (NOx and VOC), researchers analyzed the source of O_3_ and predict the change of O_3_ under different scenarios in Shanghai. Wang et al. (2001) found that the levels of major pollutants (CO, SO_2,_ and NO_x_) have increased in rural and agricultural sites in the Yangtze River Delta region of China. These pollutants are particularly evident in rural areas in North America and Europe. It is about 1–5 times the typical pollutants in rural areas of North America and Europe. The research also shows that O_3_ is positively correlated with CO and NO_x_, but the trend of positive correlation is smaller than that in rural North America. These research areas are mainly concentrated in the Beijing-Tianjin-Hebei, Yangtze River Delta, and Pearl River Delta regions. The above research has provided good support for regional O_3_ governance decisions although the methods adopted in the existing literature mainly focused on the formation mechanism of O_3_ and statistical analysis of historical data. Additionally, these studies were mostly based on large-scale spatial analysis. The sparseness of air pollution monitoring sites may be associated with the lack of research data. However, the existing spatial analysis research based on large-scale and sparse data is insufficient to support decision-making about O_3_ pollution control, especially in urban areas.

### Analysis and research on the impact of air pollution based on complex network

The complex network methodology is widely used in geography, environment, atmospheric science, information science, and finance (Gao, An & Fang, 2012; Fan et al., 2016). Numerous disciplines have set off a wave of using complex networks on research issues in their fields. Zhang & Wang (2014) constructed a network model of urban PM_2.5_ diffusion by analyzing the effects of meteorological and geographical factors. To study the fluctuation of atmospheric environmental overloading index in this region, Li et al. (2019a) built a fluctuating network based on the variation of R values in13 cities, Based on this index, they evaluated the spatiotemporal characteristics and trends of the atmospheric environmental carrying capacity (AECC). Qiang et al. (2017) studied the spatial correlation of PM_2.5_ emissions using a subnetting algorithm and proposed effective strategies to reduce PM_2.5_ pollution. Fellini et al. (2019) modelled the urban canopy as a network, for a deeper comprehension of the mechanisms that drive pollutant dispersion in urban areas. Where the streets and the intersections represent respectively the links and the nodes of the network. The direction and the weights of the links contain the geometrical characteristics of the street canyons and their wind conditions. Li et al. (2019b) on the other hand, proposed a new perspective of joint control and governance of air pollution areas based on complex networks. The outcome of the study revealed that the most cost-effective method for the prevention and control of air pollution in the Beijing-Tianjin-Hebei joint area was to control cities in the entire cluster. Xiao & Lu (2019) used a complex network to divide 68 regions in China. The research found where the main polluted (PM_2.5_) areas were located. After constructing a directional weighted network with 161 cities in China as nodes, Xue & Geng (2015) used the GN algorithm to divide the network and obtained the regional division of PM_2.5_ pollution under the influence of different seasonal factors. Ma & Gao (2018) constructed a weighted network of smog pollution between cities in the Beijing-Tianjin-Hebei region and utilized the node importance evaluation method to determine the most closely connected city network in the Beijing-Tianjin-Hebei region. Zhang et al. (2019) evaluated the impact of NO_2_ on O_3_ in time series. The researchers constructed a network of changes in Lanzhou impact degree using complex network theory and analyzed the influence of NO_2_ on O_3_ in Lanzhou.

Although a lot of research has been conducted on air pollution control using complex network methods, the research data sources are still mainly based on sparse country-based control site data. There are many limitations in using complex networks to study data on sparse country-based control sites. There is a lack of research that utilizes complex networks as well as those that focus on data from regionally intensive air quality monitoring stations.

### Research status of air quality factor O_3_ in Lanzhou

Lanzhou is located in northwestern China and the capital of Gansu Province. It is an important transportation hub city and plays an important role in the development of the western region, such as an industrial center, an economic center, and a transportation center. With the rapid economic development of Lanzhou, the acceleration of urbanization, and the rapid increase in energy consumption, Lanzhou has become one of the most polluted cities in China. Nowadays, air pollution has become an obstacle to the economic development of Lanzhou (Zhao et al., 2013; Zang, 2016).

**Figure 1:**
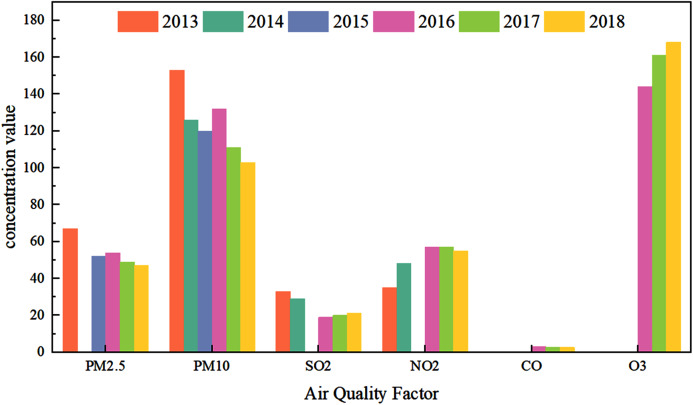
Concentration map of air quality factors in Lanzhou from 2013 to 2018.

Figure 1 shows the air quality factors PM_2.5_ (µg/m^3^), PM_10_ (µg/m^3^), SO_2_ (µg/m^3^), NO_2_ (µg/m^3^), CO (mg/m^3^), and O_3_ in Lanzhou from 2013 to 2018. (µg/m^3^) mass concentration chart. The data in the figure comes from “Atmospheric China 2019: China’s Air Pollution Control Process” (Referred to as the “Report”). According to Fig. 1, the concentration of PM pollutants generally decreased year by year whereas the concentration of O_3_ increases year by year.

The O_3_ pollution in Lanzhou has a long history. In the mid-1970s, it was the first city in China to be polluted by photochemical smog. In response to the O_3_ pollution, Lanzhou city took the lead study in the Xigu district in the late 1970s and early 1980s (Tang et al., 1989). Chen et al. (1986) studied photochemical smog in Xigu District of Lanzhou City and found that meteorological factors are important factors affecting O_3_ concentration. Other researchers (Li et al., 2018; Jiang et al., 2000) analyzed the temporal and spatial characteristics of O_3_ in Lanzhou City as well as their influencing factors. Results of the analysis showed that the concentration of O_3_ in Xigu District was the highest and its distribution had certain relationships with meteorological conditions. Lu et al. (2018) analyzed the spatial-temporal variation pattern of ozone column concentration in the Lanzhou area from 2005 to 2015 and explored new meteorological factors affecting ozone. This study also combined meteorological conditions such as sunshine and air pressure, to determine the main anthropogenic sources affecting the concentration of the ozone column.

### Research questions in this article

Generally speaking, the denser the air monitoring stations in a city are arranged, the more they can reflect the air quality in the area. Existing research is mainly based on the analysis of a very small number of state-controlled air monitoring stations in each city. This sparse monitoring station cannot cover the entire study area at all, and the sampling area is only a small part of the area, so it is difficult to precisely analyze any area. In addition, abstracting such sparse monitoring sites as a network has relatively simple structural features and poor interpretability.

Fortunately, 515 ambient air quality monitoring stations have been deployed throughout Lanzhou. The small-scale and intensive air quality monitoring stations deployed in Lanzhou provide a unique opportunity to accurately study the diffusion and transportation of atmospheric pollutants between cities and regions in the context of complex networks.

The main objective of this study is to analyze the interaction mechanism of O_3_ pollution between gridded areas in Qilihe District of Lanzhou based on data from small-scale, gridded, and dense air quality micro-monitoring stations in Qilihe District of Lanzhou.

This study takes the O_3_ hourly concentration data of dense micro air quality monitoring stations (63) in Qilihe District, Lanzhou City from November 2017 to October 2018 as the research object. This paper is mainly divided into three parts: (1) Using the complex network theory, the micro air quality monitoring sites as nodes, and the O3 network correlation network is constructed through the Pearson correlation coefficient, probability density distribution, and the maximum connected subgraph. (2) The use of the N_important method to calculate the importance ranking of the nodes, generate a directed unweighted sub-network with the strongest correlation and the highest degree of correlation among the nodes. (3) Use the transfer entropy method to calculate the transfer entropy value between the nodes of the network. This value was used as the edge weight of the subnetwork in step (2) to generate a directed weighted subnetwork. This subnetwork was used to analyze the Qilihe the mutual influence of O_3_ pollution diffusion between the various region.

## Materials

### Study areas

This study considered the Qilihe District of Lanzhou City, China as the research object. Located in the central area of Lanzhou, this area integrates housing, culture and education, small factories, and agriculture. It is a good representative for studying atmospheric pollution.

### Data sources

**Figure 2:**
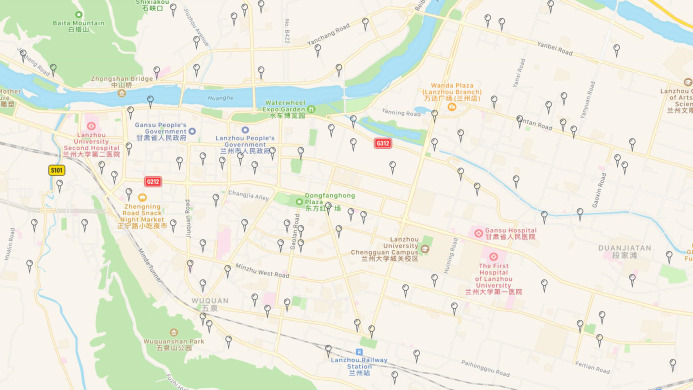
Layout of micro air quality monitoring stations in Lanzhou City (partial).

**Figure 3:**
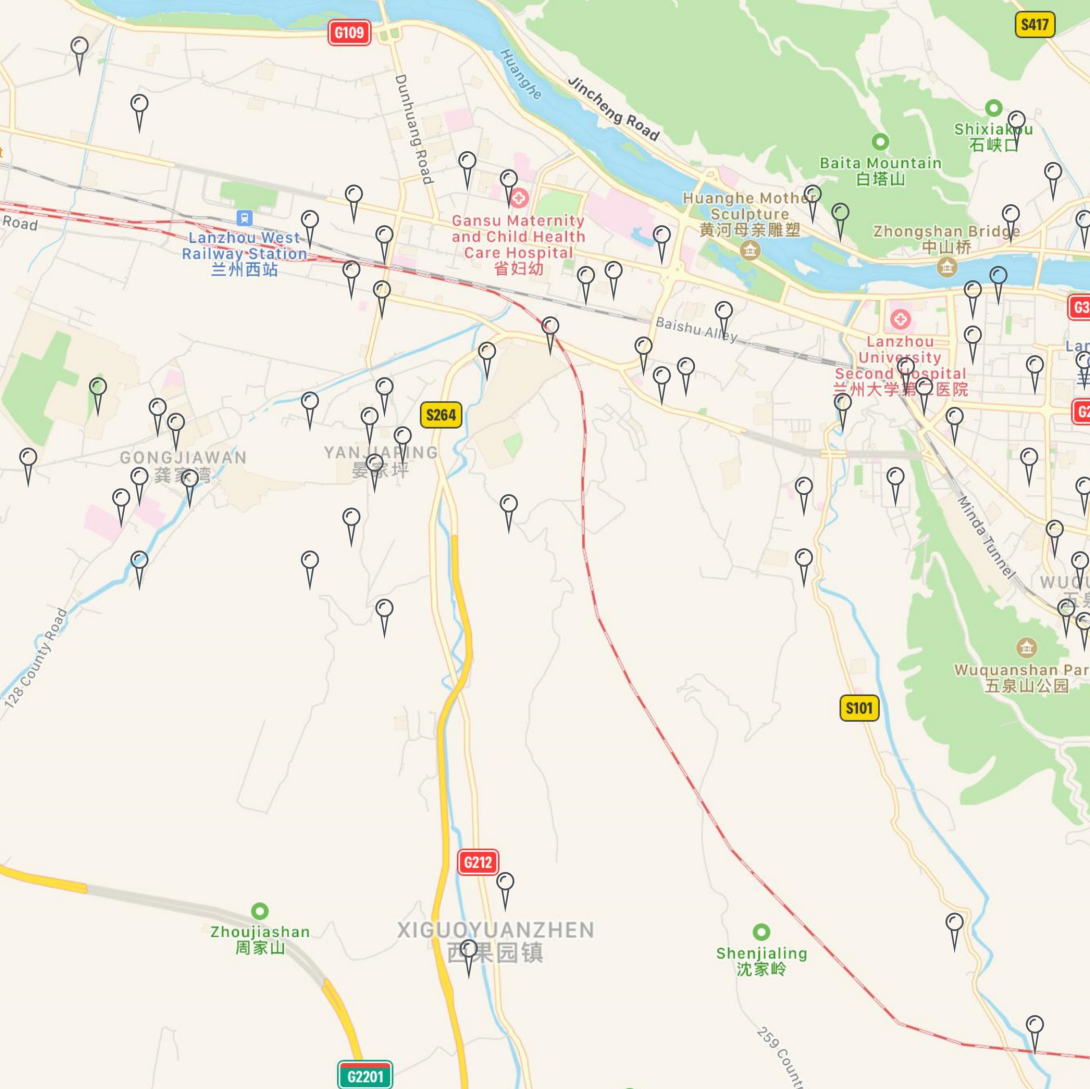
Layout of micro air quality monitoring stations in Qilihe District (partial).

**Figure 4:**
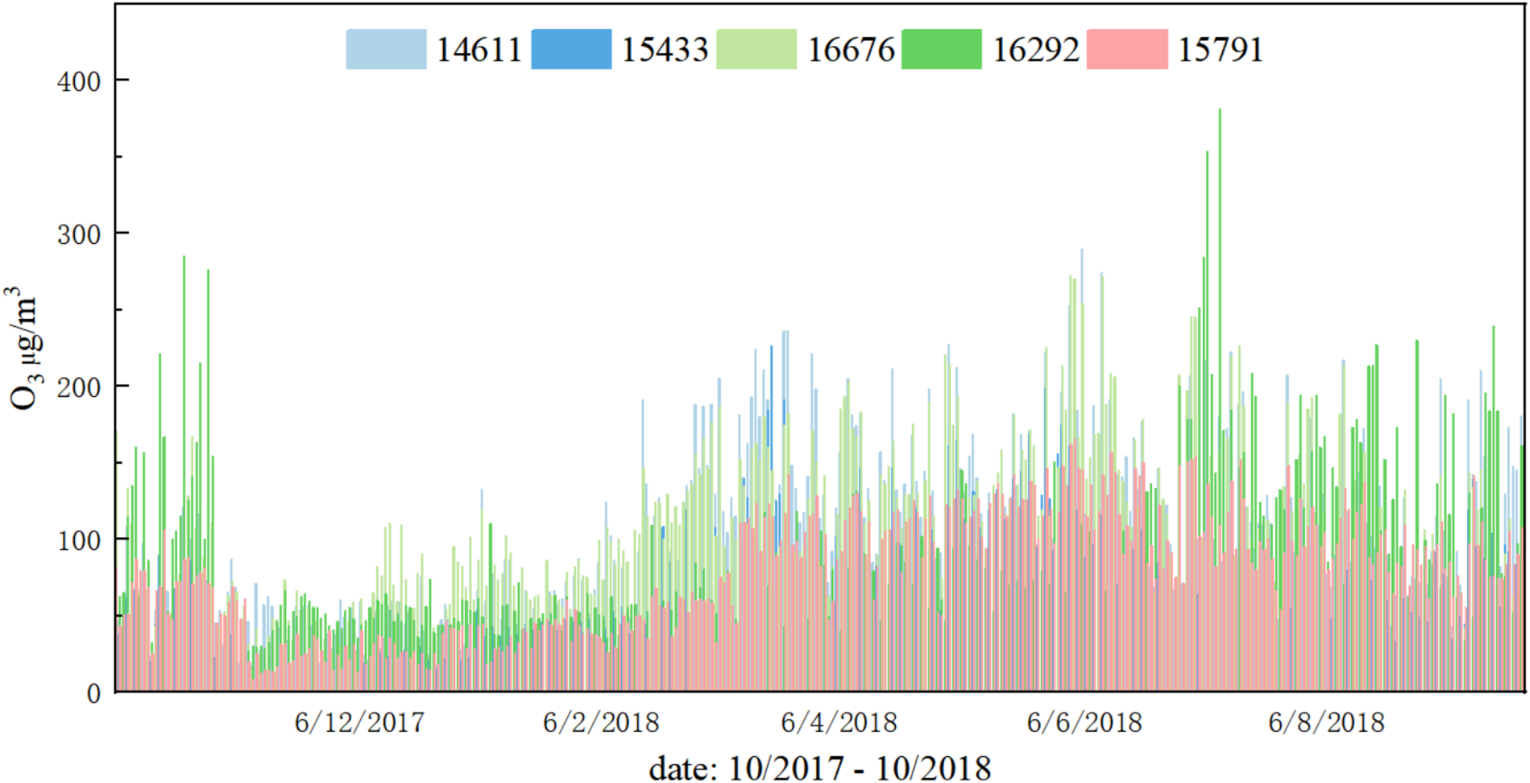
Daily changes in O_3_ concentration at five sites from November 2017 to October 2018.

Lanzhou deployed micro stations that monitor air quality, as shown in Fig. 2. The micro stations for ambient air quality deployed in Qilihe District are screened out from the micro stations (See Fig. 3). This study uses the monitoring data set of the dense air quality monitoring station deployed in Qilihe District, Lanzhou City, and selects a data set of air quality factor O_3_ according to the air quality data specifications and standards of ‘Environmental Air Quality Standard (GB3095-2012)’. After excluding sites with serious defects in data volume, 63 air quality monitoring stations were selected that could cover the Qilihe District. When the continuous data gap measurement within a day is greater than 4 h, the day will be eliminated. When the data deficiency measurement in a day is less than or equal to 4 h, the average value of the upper and lower two data closest to the deficiency measurement value in the day will be used to fill the deficiency measurement value. Through processing, hourly concentration data of O_3_ without missing values at 63 stations in Qilihe District from November 2017 to October 2018 were obtained, each site has obtained 5,259 pieces of data respectively, and the final research data totals 331,317 pieces. This paper randomly selected five micro-sites to display the daily changes of ozone concentration, as shown in Fig. 4.

## Construction of Spatial Correlation Network of Interregional O_3_ Pollution

### Edge of network

#### Calculation of correlation of O_3_ time series between stations

**Figure 5:**
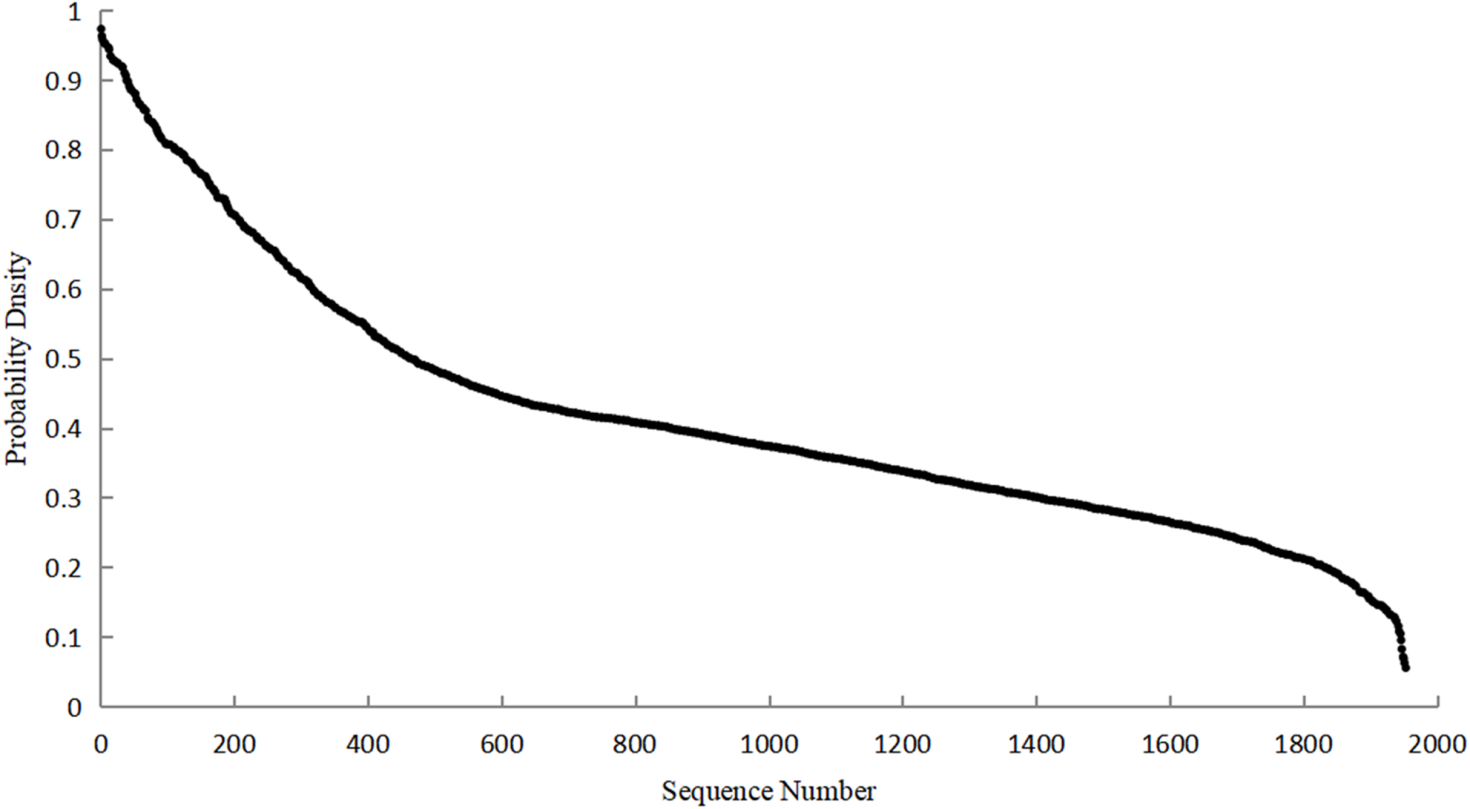
Correlation coefficient distribution.

By calculating the ozone Pearson correlation of any two air monitoring stations on the time series, the correlation degree between the stations is obtained (Zhang et al., 2019), and the distribution of the correlation coefficient is as follows:

Figure 5 shows that the distribution curve of the correlation coefficient first shows a largely downward trend. When the correlation coefficient is greater than or equal to 0.5, the downward trend starts to decrease. The above pattern indicates that the weaker relationship is the majority whereas the stronger one is the minority. Most of the correlation coefficients are distributed between 0–0.5 while on few are distributed between 0.5–1.

#### Selection of the optimal connection threshold of network edge

If the selected Pearson correlation coefficient, the threshold is too small, the weak correlation nodes are also linked, but if the threshold is too large, some valuable edges may be filtered out. Therefore, it is necessary to select the optimal connection threshold (Fan et al., 2016) for the network.

**Figure 6:**
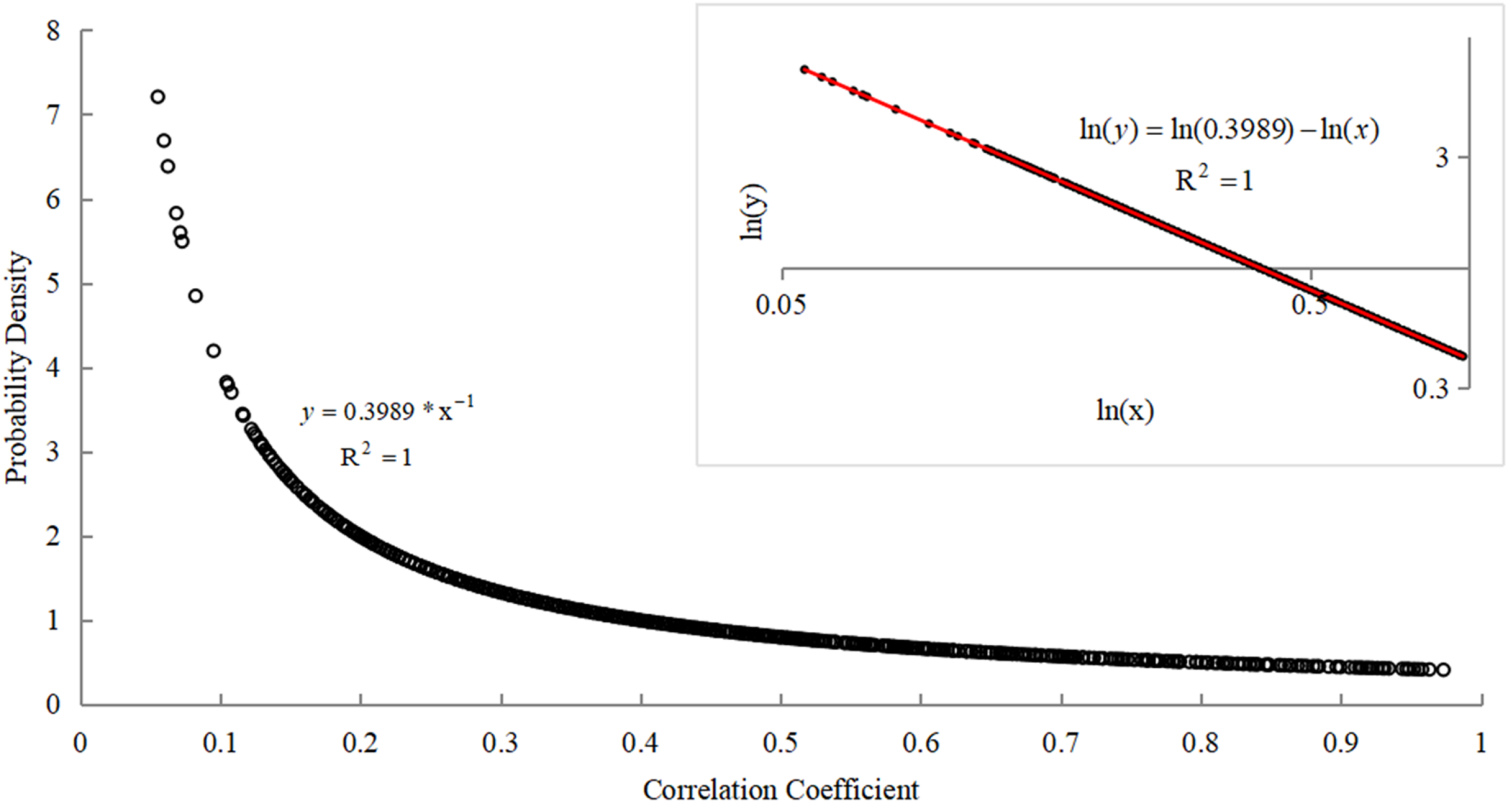
Probability density distribution map of correlation coefficient between ambient air quality detection stations.

The construction of the optimal spatial correlation network is closely related to the selection of the optimal connection thresholds that form the edge of the network (Fan et al., 2016). The selection of the optimal connection threshold of network nodes is closely related to the probability density distribution of the correlation coefficients between the ambient air quality monitoring sites. The probability density distribution diagram of the correlation coefficient can be plotted as shown in Fig. 6. The big picture in this figure is a scatter plot of the probability density distribution of the correlation coefficient between the ambient air quality monitoring sites. The least-square method was used to fit it and the fitting relationship was: $y = 0.3989*{x^{ - 1}}$. The goodness of fit is: ${R^2} = 1$. The small picture is formed by taking the log of the double coordinate system of the big picture and fitting the log scatter plot by the least square method. It was found that the effect after the fitting completely obeys the linear relationship. The expression of the linear relationship was obtained as: ${\rm ln(}y) = \ln (0.3989) - \ln (x)$ and the goodness of fit was: ${R^2} = 1$. The probability density distribution function was: $y = 0.3989*{x^{ - 1}}$.

As shown in Fig. 6, most of the correlation coefficient values are small and only a few correlation coefficient values are large. Therefore, according to the characteristics of power-law distribution, the strong correlation coefficient selected in this study was used as the connected edge of the network. Based on the calculations, the critical value of the strong correlation coefficient and the weak correlation coefficient was 0.536619. That is, the value range of the correlation coefficient is $[0.536619 - 1)$ and the value range of the correlation coefficient was $(0 - 0.536619)$. The networks constructed based on different thresholds (correlation coefficient) are different. They have different maximum connected subgraphs (Wu & Li, 2008). The number of nodes in the maximum connected subgraph (MCSG) can affect the stability of the network.

**Figure 7:**
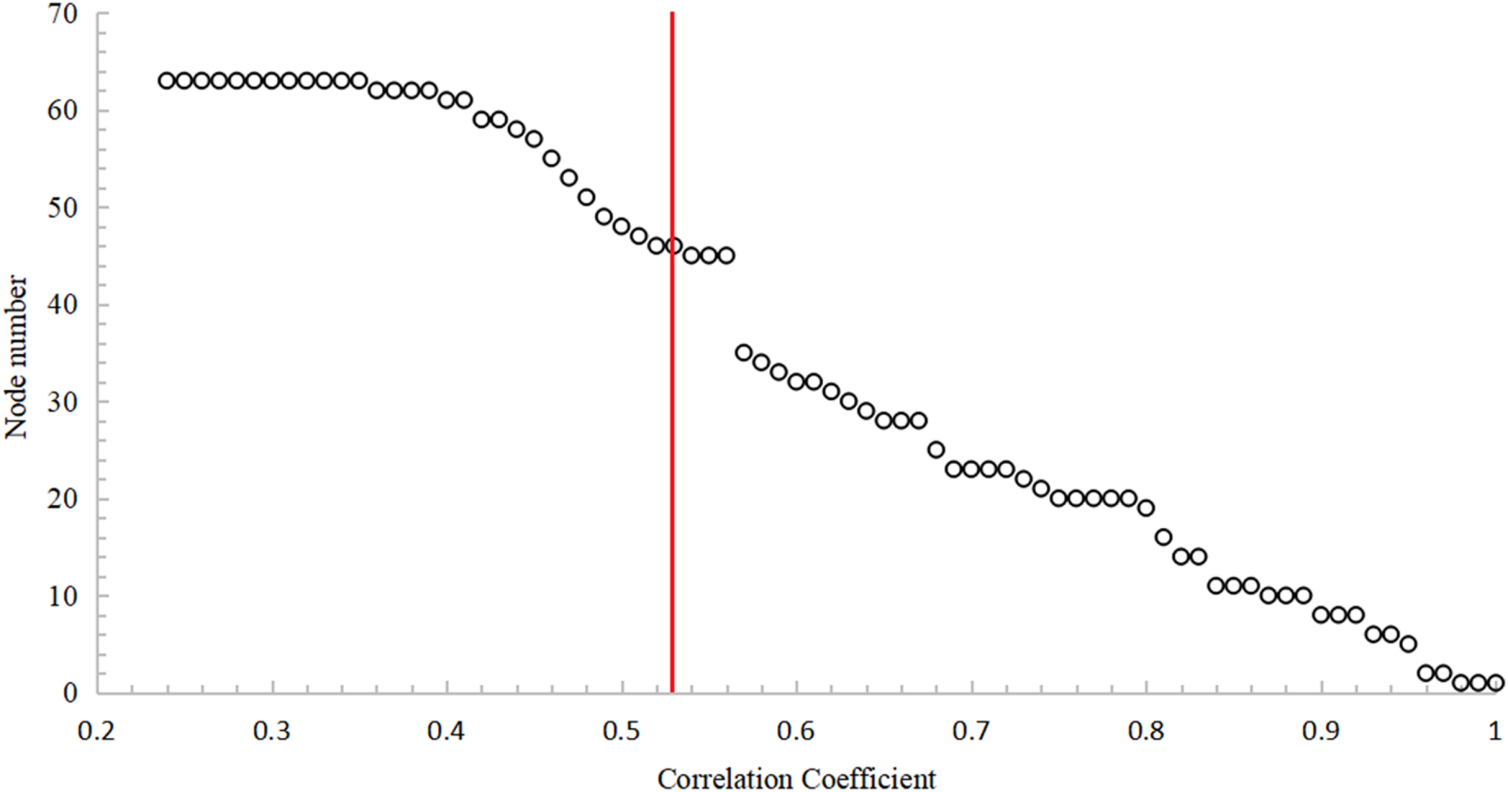
The trend of the number of nodes in maximum connected subgraph with the correlation coefficient.

It can be found from Fig. 7 that as the correlation coefficient increases, the number of nodes in the network’s maximum connected subgraphs shows a gradual decrease. When the correlation coefficient is between [0.49;0.56], the change in the number of nodes is relatively stable, indicating that this interval can be used as the optimal connection threshold interval for the network.

Based on the analysis of the correlation coefficient probability density distribution and the MCSG, 0.536619 was selected as the optimal connection threshold value of the network nodes. The red vertical line in Fig. 7 represents the position of the abscissa which is 0.536619. If the correlation coefficient between two nodes is greater than or equal to 0.536619, there is an edge between the two nodes and vice versa.

### Network topology

**Figure 8:**
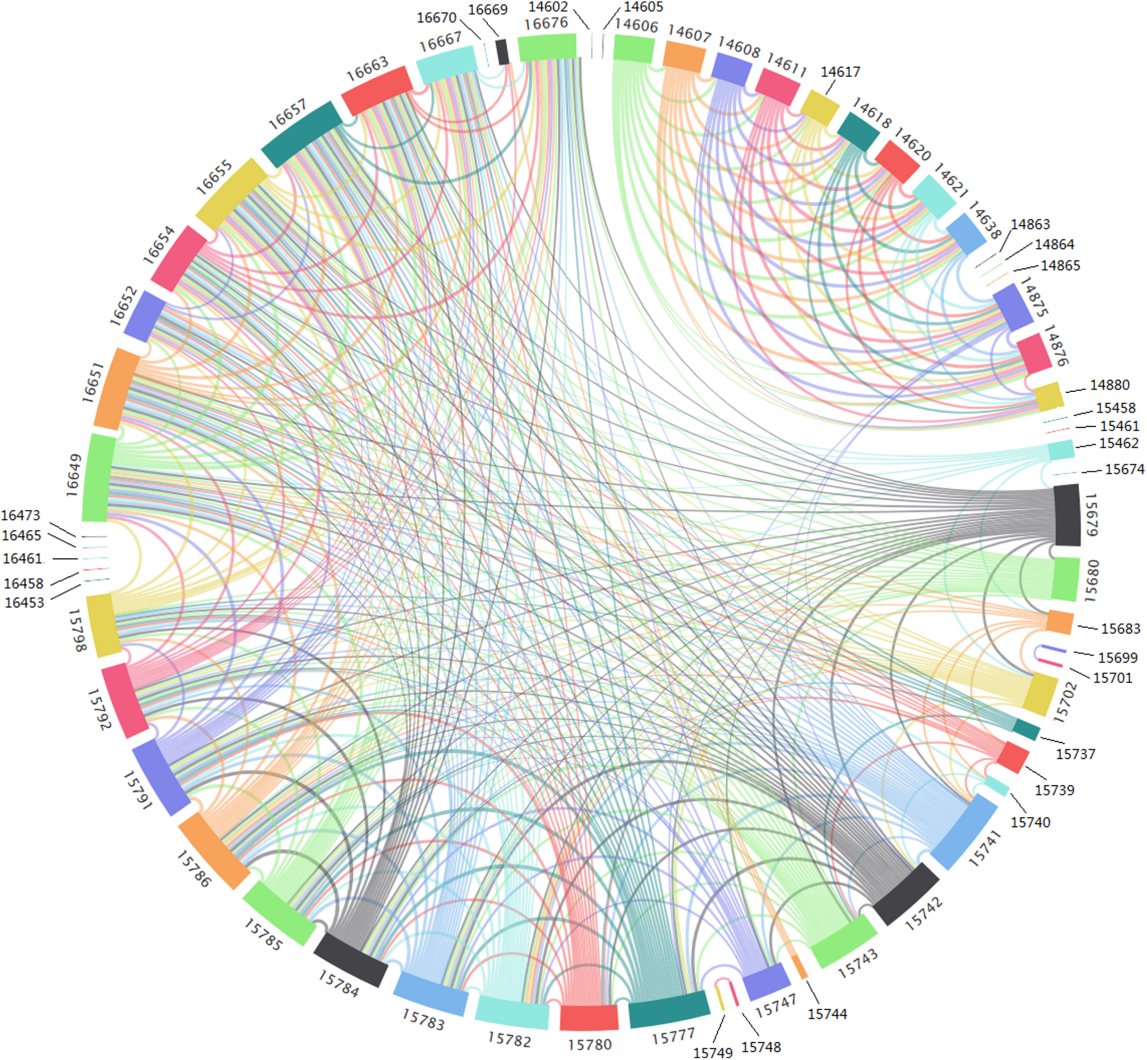
Spatial correlation network topology.

Under the optimal connection threshold of the network nodes of 0.536619, the relationship network of ambient air quality monitoring stations in Qilihe District, Lanzhou City, a total of 63 nodes and 408 edges were generated as shown in Fig. 8.

The rectangle-shaped colored filled box in Fig. 8 is a network node, the number (14,605) outside the colored filled box is the label of the network node and the colored lines between the colored filled boxes are connected edges between the nodes. The color of the edges is not considered in Fig. 8 and the edges in the figure are all undirected and unweighted. The longer the color-filled box, the greater the number of nodes with a higher correlation with the nodes represented by the color-filled box. The reality indicates that there are more stations with similarities about the O_3_ pollution level and O_3_ concentration change trend of the ambient air quality monitoring station and vice versa. Figure 8 also shows that most nodes have connected edges while individual nodes are isolated and have no connected edge with any other node. This indicates that the air quality monitoring sites represented by these isolated nodes are comparable with other sites. The correlation between the degree of O_3_ pollution and the trend of O_3_ concentration was low or almost showed non-correlation. Therefore, the mutual influence relationship between the nodes cannot be found from these isolated nodes and there is independence in the change of O_3_ mass concentration. These results indicate that O_3_ pollution of these nodes cannot be achieved using joint management between nodes but rather, a single governance measure should be developed and adopted.

According to the spatial correlation network topology characteristic map of the O_3_ pollution between regions (Fig. 8), the area represented by 63 nodes can be divided into three parts from the connection on the network edge. The first part is mainly distributed in the northern part of the Qilihe District. The nodes represent the community area of urban streets. Human activities are frequent and complex in the first part and the monitoring stations are densely distributed. It is therefore expected that the O_3_ concentration generated from air quality monitoring stations in this area will be influenced by several factors. The impact caused the O_3_ pollution degree and O_3_ concentration change law to be very different and the correlation degree was reduced. The second part is mainly distributed in the mountainous area in the south of Qilihe District. The nodes represent township and village-type areas. Additionally, human activities are relatively rare in these areas. The sparse distribution has fewer or single factors affecting the O_3_ concentration of the air quality monitoring stations in the area. This makes the degree of O_3_ pollution and O_3_ concentration change law to have high similarities. The third part is the area represented by isolated nodes. This part of the area is relatively scattered, which is not representative.

## Node Importance Analysis of Spatial Correlation Network of Interregional O_3_ Pollution

### Node importance evaluation index and algorithm

Some common classic node importance evaluation indicators and algorithms reported in the literature include; degree, degree centrality (Zhao et al., 2013), Closnesscentrality (Guan, Li & Xing, 2019; Freeman, 1979), betweenness (Freeman, 1997; Brandes, 2001), Pagerank algorithm and its derivatives (He & Li, 2011; Page et al., 1999; Liu et al., 2018; Brin & Page, 1998).

The above-mentioned node importance evaluation indexes and algorithms have their limitations. For instance, the probability of a node randomly walking to other nodes in the Pagerank algorithm is the same and cannot reflect the importance of the nodes. This paper refers to the Pagerank optimization algorithm in He & Li (2011), which uses the edge weight matrix of the spatial correlation network to replace the random walk transition probability matrix in the Pagerank algorithm and uses the Closnesscentrality matrix of node degree to replace the probability matrix (uniformly distributed parameter) of PageRank algorithm which starts from the node $i$ and stays on the node $i$.

The core formula of the Pagerank algorithm is as follows (1):

(1) $$P{R_i} = p*\sum\limits_{j \in {M_i}} {\displaystyle{1 \over {{L_j}}}} *P{R_j} + (1 - p)*\displaystyle{1 \over n}$$

Among them, ${\rm PR}(i)$ and ${\rm PR}(j)$ are the probability that web pages $i$ and $j$ are visited; $p$ is the damping coefficient; ${M_i}$ is the set of web pages that have links to page $i$; ${L_j}$ is the number of other web pages that are accessed by a web page $j$; $n$ is the total number of web pages.

This study analyzes the shortcomings of the Pagerank algorithm and modifies it based on the adjacency matrix, weight matrix, and related network topology features of the completed network. This makes the modified algorithm more suitable for finding important nodes in this study:

1) According to the O_3_ mass concentration time series data of the air quality monitoring station. If the correlation coefficient is $cor({v_i },{v_j }) > 0.536619$, then the corresponding element in the matrix is set to 1, otherwise, the corresponding element in the matrix is set to 0 to generate the adjacency matrix $Adj\_m$ of the network. The specific operation is shown in Eq. (2).

(2) $$Adj\_m(i,j) = \left\{ \matrix{1  ({v_i },{v_j }) \in G \; {\rm and} \;cor({v_i },{v_j }) \gt 0.536619 \cr 0  ({v_i },{v_j })\ \notin\ G \;{\rm and} \;cor({v_i },{v_j }) \le 0.536619 } \right.$$

Among them, $cor({v_i },{v_j })$ is the correlation coefficient of the O_3_ time series on the air quality monitoring station represented by nodes $i$ and $j$ in the spatial correlation network. $Adj\_m(i,j)$ is a value where the position of the adjacency matrix $Adj\_m$ is $(i,j)$; G is a network; to remove self-connections in the network, the elements on the diagonal of the adjacency matrix are all 0.

2) Since only the case where there are connected edges between two nodes in the network is discussed, the adjacency matrix of the network is used to process the correlation matrix between the nodes to obtain the edge weight matrix $New\_Cor\_S$ of the network. The specific operation is shown in Eq. (3).

(3) $$New\_Cor\_{S_{(i,j)}} = Cor\_{S_{(i,j)}}*Adj\_{m_{(i,j)}}$$

Among them, $New\_Cor\_{S_{(i,j)}}$, $Adj\_{m_{(i,j)}}$ and ${\rm Cor}\_{S_{(i,j)}}$ represents the elements at positions $(i,j)$ in the matrices ${\rm New\_Cor}\_S$, $Adj\_m$, and ${\rm Cor}\_S$.

3) The transition probability matrix $Random \_m$ (Eq. 4) based on the correlation between nodes is used instead of the Pagerank algorithm and the transition probability matrix.

(4) $$Random \_{m_{(i,j)}} = \displaystyle{{New\_{\rm Cor}\_{S_{{\rm (i,j)}}}} \over {\sum\nolimits_{i = 1}^n {New\_{\rm Cor}\_{S_{{\rm (i,j)}}}} }}$$

Among them, $\sum\nolimits_{i = 1}^n {New2\_{\rm Cor}\_{S_{{\rm (i,j)}}}}$ is the sum of the elements in the column $j$.

4) According to the constructed network, the node degree can reflect the importance of the node in the network, the greater the degree of a node, the higher the node’s Closnesscentrality. In this study, the Closnesscentrality matrix was selected to replace the probability (uniformly distributed parameter) matrix $Pro\_m$ that randomly walks from the node $i$ but resides on the node $i$ in the network. The specific operations are shown in Eqs. (5), (6).

(5) $$CC({v_i}) = \displaystyle{1 \over {\overline D }}$$

(6) $$C{C_{(1*n)}} = [CC({v_1}),CC({v_2}), \cdots ,CC({v_i}), \cdots ,CC({v_n })]$$

Among them, $C{C_{(1*n)}}$ is the Closnesscentrality matrix of order $1*n$; $CC({v_j})$ is the Closnesscentrality of the node $i$; $\overline D$ is the average shortest path from the node $i$ to other nodes.

5) According to the definition of the random matrix and the conditions that are satisfied, let $New\_CC$ be a non-negative random matrix of order $1*n$, and $CC({v_i})$ be the element at the $(1,i)$ position in the matrix, then $\forall i = 1,2, \cdots ,n$, $CC({v_i}) \ge 0$, and $\sum\nolimits_{i = 1}^n {CC({v_i})} = 1$ are satisfied; Since the process of randomly walking from a node to its neighbors is independent of time, therefore, then the random walk process is a finite homogeneous Markov process, and the random matrix $New\_CC$ is called the transition probability matrix of the Markov process. The specific operation is as shown in Eq. (7).

(7) $$New\_CC = \displaystyle{{C{C_{(1*n)}}} \over {\sum\nolimits_{i = 1}^n {CC({v_i})} }}$$

6) Using the random walk model, the node importance formula was obtained and named the N_important method. The specific operation is shown in Eq. (8).

(8) $$N\_importanc{e_{(n*1)}} = p *{\rm Random\_m}*N\_importanc{e_{(n*1)}} + (1 - p)*New\_CC$$

By calculating Eq. (8), the process is as follows and Eq. (9) is finally obtained.

$\Rightarrow$ $({\rm E - p} *{\rm Random\_m})*N\_importanc{e_{(n*1)}} = (1 - p)*New\_CC$

$\Rightarrow$ ${(N\_importanc{e_{(n*1)}})^{\rm T} }*{({\rm E - p} *{\rm Random\_m})^{\rm T} } = (1 - p)*New\_CC$

$\Rightarrow$ ${(N\_importanc{e_{(n*1)}})^{\rm T} } = (1 - {\rm p})*New\_CC*{({({\rm E - p} *{\rm Random\_m})^{\rm T} })^{ - 1}}$

(9) $$N\_importanc{e_{({\rm n*1})}} = {((1 - {\rm p})*New\_CC*{({({\rm E - p} *{\rm Random\_m})^{\rm T} })^{ - 1}})^{\rm T}}$$

Among them, $N\_importanc{e_{(n*1)}}$ is the $n*1$ order node importance matrix; $p *{\rm Random\_m}*N\_importanc{e_{(n*1)}}$ indicates the probability of random walk from a node (whether the node is $i$ or not) to other nodes and then randomly walk to the node $i$ again; $(1 - p)*New\_CC$ represents the probability of random walk from node $i$ to stay on the node $i$; $E$ is the unit matrix.

7) From the node importance formula in step 6, information about the probability of a node being visited is controlled by its probability of being visited becomes evident. To avoid the situation where the importance of the nodes of the network is all 0 when $p = 1$ and the situation where the importance of the nodes of the network is only controlled by the betweenness when $p = 0$, the value of $p$ should be between $\left( {0,1} \right)$. According to the general value of $p$, when $p = 0.85$, the algorithm works best. Therefore, the value of $p$ in this study is 0.85.

### Comparative analysis of node importance results

According to the network constructed above, the node importance evaluation methods such as Degree (DC), Closnesscentrality (CC), Pagerank (PR), and the N_important algorithm optimized in “Node Importance Evaluation Index and Algorithm” are used to calculate the node importance ranking results of each method. The top 10 nodes in each method were selected and shown in Table 1.

**Table 1:** Sort results calculated by the node importance evaluation algorithm (top 10 nodes).

Sequence Number	N_Important	Pagerank	Closnesscentrality	Degree
1	16649	16649	16649	16649
2	16657	15741	***15741***	***15741***
3	15786	***16655***	***15786***	***15786***
4	16655	***16657***	***16655***	***16655***
5	16651	15786	***16657***	***16657***
6	15741	16651	15782	15782
7	15777	15782	16651	16651
8	15791	15792	15777	15777
9	15785	15777	15792	15792
10	15784	15742	15742	15742

**Note:**

“16649” in table represents the labels of the nodes. The data in bold in the italics are the labels of the nodes with the same importance degree.

Table 1 shows that the N_important method can reflect the strength of the nodes, while the importance index values of some nodes in the other three methods are equal and cannot reflect the strength of the nodes. The CC method is the same as the DC method in the top 10 nodes. In these two methods, the value of nodes 15741 and 16657 is equal, and the difference between the two nodes cannot be judged. However, in the N_important method, the value of node 16657 is greater than node 15741. Thus, node 16657 is placed in front of node 15741. This is because, in the transition probability matrix of the N_important method, the probability of a random walk from a node to node 16657 (average probability is 0.33) is greater than the probability of a random walk from a node to node 15741 (average probability is 0.31). In the N_important method, node 16657 is ranked before node 15741. This is mainly because the coefficient of the transition probability matrix in Eq. (10) is larger than the coefficient of the uniformly distributed parameter. The situation for nodes 16657 and 15786 is the same as the above analysis. Compared with the N_important method, the node rank ordering of the Pagerank method is very different. For nodes 16655 and 16657, the probability of random walk from one node to node is equal. and in the uniformly distributed parameter matrix, random walks between nodes without priority. The above analysis shows that the N_important method can avoid the shortcomings of the PageRank method and is more effective in sorting the importance of nodes.

### N_important method result analysis

In the spatial correlation network, each node label represents a realistic area. For example, the ambient air quality monitoring site represented by node 15741 is deployed in Guoyuan Village, Bali Town, Qilihe District Lanzhou City. According to the analysis of the importance ranking results of the above nodes, it is quite evident that the node importance results of the N_important method have more advantages. Therefore, the following research in this study focuses on the results of node importance calculated by the N_important method. The top 10 nodes and the real regions they represent are shown in Table 2.

**Table 2:** The top 10 nodes of node importance and the real area they represent.

Sequence Number	Node Label	Real Area
1	16649	Qinggang Village, Xiguoyuan Town, Qilihe District (2215)
2	16657	Xiguoyuan Village, Xiguoyuan Town, Qilihe District (2256)
3	15786	Gaolingou Community, Agan Town, Qilihe District (1619)
4	16655	Shangguoyuan Village, Xiguoyuan Town, Qilihe District (2222)
5	16651	Yuanjiawan Village, Xiguoyuan Town, Qilihe District (1915)
6	15741	Guoyuan Village, Bali Town, Qilihe District (1644)
7	15777	Lannigou Community, Agan Town, Qilihe District (1681)
8	15791	Dashuizi Community, Agan Town, Qilihe District (1550)
9	15785	Pingling Village, Agan Town, Qilihe District (1595)
10	15784	Gaolingou Community, Agan Town, Qilihe District (1651)

**Note:**

The values in parentheses after the realistic areas in table are the numbers of the ambient air quality monitoring stations. There may be several monitoring stations in an area, such as Gaolingou Community (1619) in Agan Town, Qilihe District and Agan in Qilihe District Town Gaolingou Community (1651).

Table 2 shows the top 10 nodes of the N_important method and the actual regions they represent. These 10 nodes are mainly distributed in Agan Town, Xiguoyuan Town, and Bali Town. These areas are located in the southeast of the Qilihe District and are geographically adjacent to each other. The inter-regional correlation is strong and the extent of pollutant diffusion and transport between regions are also strong. As a result, the risk of O_3_ pollution in these areas is quite high. The degree and the changing trend of O_3_ mass concentration have high similarities.

## Analysis of the Spatial Interaction of O_3_

To further discuss the mutual influence of O_3_ pollutants in adjacent areas, the nodes with the highest node importance are extracted from the original spatial correlation network to form a sub-network with the strongest correlation and highest correlation among the nodes.

**Figure 9:**
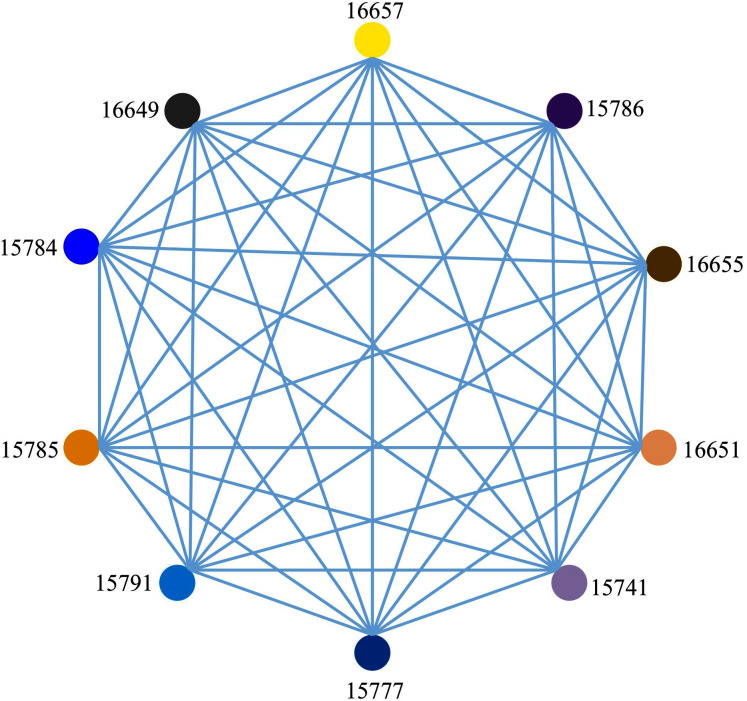
Adjacent area spatial correlation network.

Figure 9 is a fully connected adjacent region spatial correlation network with 10 nodes and 45 edges constructed under the condition of the optimal connection threshold of the original spatial correlation network of 0.536619. In this network, there are connecting edges between two nodes, indicating that the connection and correlation between any two nodes are strong. It can be seen from Fig. 9 that each node has an influence on each other but the direction and degree of mutual influence between nodes cannot be determined. Therefore, a causality detection method is used to determine the direction and degree of mutual influence between nodes.

### Transfer entropy

To accurately determine the direction and extent of ozone interaction between regions, it is necessary to conduct causality tests on the ozone concentration sequence of each site. Granger causality is generally used in causality testing. The main idea of this method is to consider two random variables X and Y. If X is predicted when the value of Y is added, the prediction effect of X can be improved, that is, Y has a causal effect on X, However, this method does not consider the influence of interference factors, nor the nonlinear interrelationship between time series, so the causality in nonlinear complex systems can no longer be tested with Granger causality. Transfer entropy can detect causality between non-linear time series. It is a nonlinear method that uses the Markov property to detect information transmission and deduce the direction of causality. The air quality time series in this paper is nonlinear. Therefore, we use transfer entropy to calculate the information transfer between air quality time series. At this time, the information transfer between air quality time series can be compared to the mutual diffusion of O_3_ between air quality monitoring stations. For the time series X and Y of two micro stations, the interaction relationship between the two regions is discussed by calculating the transfer entropy of the two sequences. Suppose the O_3_ mass concentration time series of the two air quality monitoring stations are $X$ and $Y$, $x \in X,\; y \in Y$, the discrete distribution probabilities of the two-time series are $P(x)$ and $P(y)$, the joint probability is $P(x,y)$ and the conditional probability is $P(x|y)$. The calculation process of transfer entropy between air quality monitoring stations is as follows:

Based on Kullback–Leibler entropy (Kullback & Leibler, 1951), Thomas Schreiber (Kaiser & Schreiber, 2002) defines the transfer entropy as shown in Eq. (10).

(10) $${T _{y \to x}} = \sum {P(\mathop x\nolimits_{n + t} ,\mathop x\nolimits_n^m ,\mathop y\nolimits_n^l )} \log \displaystyle{{P(\mathop x\nolimits_{n + t} |\mathop x\nolimits_n^m ,\mathop y\nolimits_n^l )} \over {P(\mathop x\nolimits_{n + t} |\mathop x\nolimits_n^m )}}$$

In Eq. (10), ${T _{y \to x}}$ represents the amount of information transmitted by $Y$ to $X$, $m$ and $l$ represents the length of the time series under the effect of the delay time; Where ${x_n}$ is the value of a sequence $X$ at a time $n$ and ${y_{n - t}}$ is the value of a sequence $Y$ at a time $n - t$. $\mathop x\nolimits_n^m = (\mathop x\nolimits_n ,\mathop x\nolimits_{n + 1} , \cdots ,\mathop x\nolimits_{n - m + 1} )$; $P(\mathop x\nolimits_{n + t} ,\mathop x\nolimits_n^m ,\mathop y\nolimits_n^l )$ represents the joint probability distribution from $(\mathop x\nolimits_n^m ,\mathop y\nolimits_n^l ) \to \mathop x\nolimits_{n + t}$; $P(\mathop x\nolimits_{n + t} |\mathop x\nolimits_n^m ,\mathop y\nolimits_n^l )$ represents the conditional probability from $(\mathop x\nolimits_n^m ,\mathop y\nolimits_n^l ) \to \mathop x\nolimits_{n + t}$ Distribution; $P(\mathop x\nolimits_{n + t} |\mathop x\nolimits_n^m )$ represents the conditional probability distribution from $\mathop x\nolimits_n^m \to \mathop x\nolimits_{n + t}$; the delay time from the state $\mathop x\nolimits_n^m \to \mathop x\nolimits_{n + t}$ is $t$, that is, $m = t$.

### Interaction analysis of O_3_ space based on transfer entropy

The O_3_ time series data of each monitoring station obtained above was used to calculate the transfer entropy value between the nodes. As shown in Table 3, the numbers in bold the table are the network nodes. Based on Fig. 8, a directed weighted network with 10 nodes and 90 edges was generated using the entropy value as the edge weight of the network, as shown in Fig. 9. The color of the edges is considered in Fig. 9 and the edges in the figure are all directed weighted edges. Among them, starting from a certain (such as X color-filled frame) color-filled frame, a color line with the same color as (X) color-filled frame represents the out-degree of the spatial correlation network of adjacent areas, and in reality, represents the impact of diffusion and transportation on other areas of the O_3_ pollution in that area. Colored lines with different colors of starting from other colored filled boxes to (X) colored filled frames, representing the entry-degree of the spatial correlation network of adjacent areas. In reality, it means the impact of O_3_ pollution in other areas on the Area represented by (X) filled frame through diffusion and transport. The wider the color line, the higher the degree of influence of O_3_ between regions.

**Table 3:** Transfer entropy values between nodes (the edge weights of network).

	16649	16657	15786	16655	16651	15741	15777	15791	15785	15784
**16649**	0	0.188165	0.416685	0.165089	0.252276	0.444452	0.40793	0.399503	0.336574	0.37785
**16657**	0.200926	0	0.386019	0.160254	0.228713	0.402894	0.364544	0.360518	0.308879	0.360223
**15786**	0.302260	0.277764	0	0.244699	0.302192	0.378277	0.205059	0.184778	0.158459	0.180941
**16655**	0.230554	0.181400	0.351521	0	0.248165	0.381882	0.354523	0.338460	0.278343	0.331844
**16651**	0.298259	0.234665	0.368420	0.231545	0	0.382172	0.339363	0.354950	0.309469	0.332464
**15741**	0.362590	0.325377	0.297446	0.287788	0.355317	0	0.267426	0.297404	0.232480	0.258994
**15777**	0.316643	0.296403	0.207337	0.258552	0.306774	0.371639	0	0.218252	0.161476	0.154222
**15791**	0.304913	0.269050	0.199517	0.250782	0.299133	0.372282	0.204188	0	0.173749	0.206157
**15785**	0.302496	0.273973	0.233110	0.227895	0.31673	0.371759	0.193099	0.224856	0	0.195817
**15784**	0.295051	0.266683	0.170130	0.245908	0.281904	0.341574	0.149102	0.196428	0.137239	0

**Figure 10:**
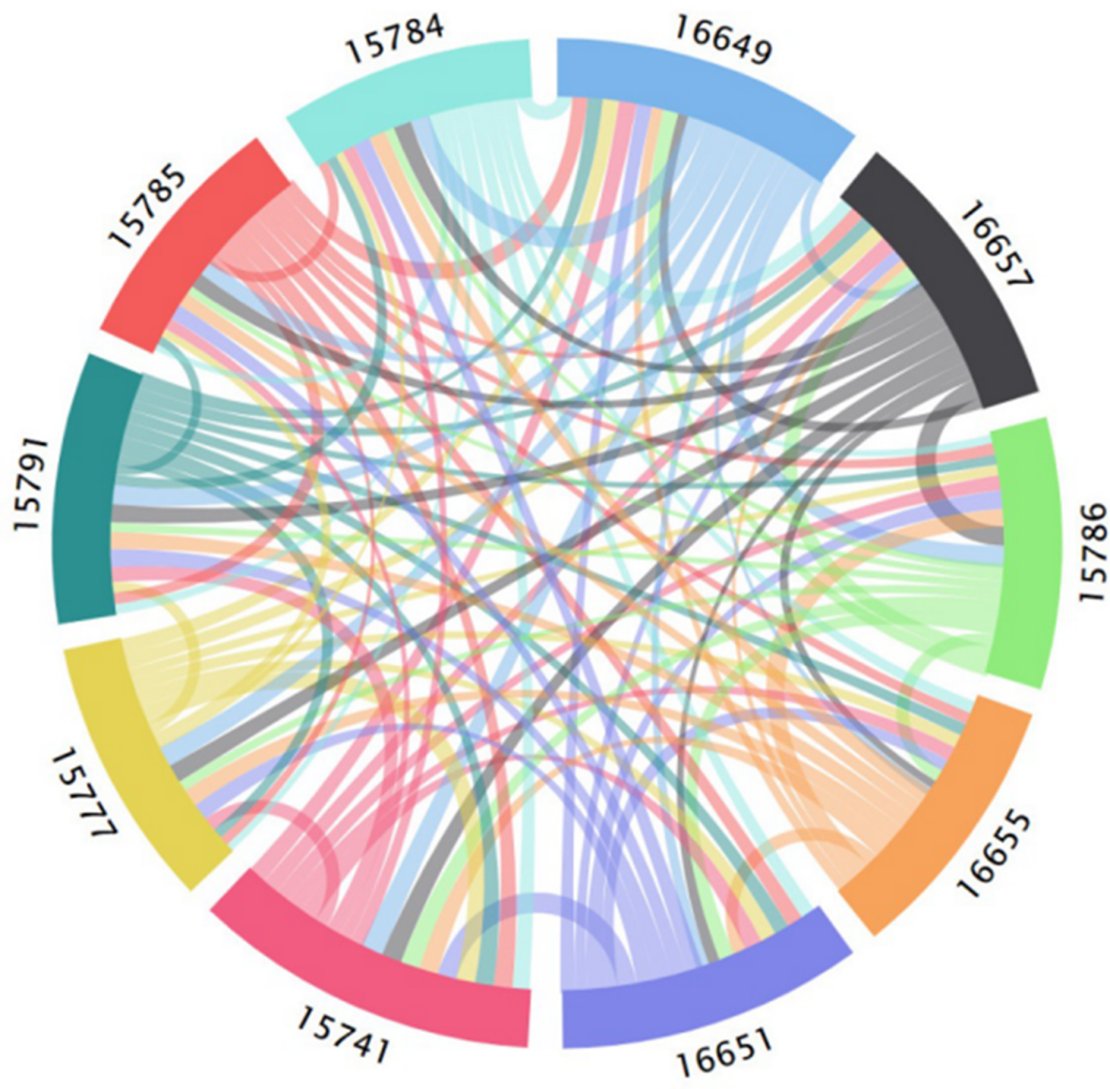
Spatial correlation network of adjacent regions based on transfer entropy.

As can be seen from Table 3 and Fig. 10, the node identified by the underlined data 15741 (Guoliyuan Village (1644), Bali Town, Qilihe District) is most affected by O_3_ pollution from other nodes. Among them, node 16649 (Xiguoyuan Town, Qilihe District) Qinggang Village (2215)) has the greatest impact on node 15741. 15785 (Pingling village, Agan Town, Qilihe district (1595)) is the weakest node affected by O_3_ pollution of other nodes of which 15784 (Gaolinggou community, Agan Town, Qilihe district (1651)) has the least impact on 15785. Under the conditions with the greatest degree of influence, the most scope of influence area is node 16649. To control the O_3_ pollution of node 15741, it is necessary to start with other nodes that affect the node and eliminate them from the source. For node 15785, we must proceed from ourselves and take measures according to the actual situation. For node 16649, the actual area should be treated as a key target. Results in Table 3 and Fig. 10 indicate that the actual area represented by each node will be affected by O_3_ pollution between each other. However, due to the different degrees of influence between each area, the conditions that cause these effects are also different in each area. Therefore, these areas cannot be used as a whole for the treatment of O_3_ pollution. To address O_3_ pollution in the study area, a cooperative governance mechanism between adjacent areas should be established to achieve joint control and governance of regional O_3_ pollution. Furthermore, there is the need to implement an O_3_ pollution warning mechanism and establish emergency measures to reduce the emissions of O_3_ prerequisites (NO_x_ and VOCs).

## Discussion and Conclusions

### Discussion

Compared with most previous works in the literature, this work is characterized by large-scale micro-monitoring sites data. These data provide us with the possibility to accurately explore the interaction mechanism of small-scale pollutants. Most of the known research on air pollution is mainly based on the results obtained from the air quality monitoring station controlled by the state. These monitoring sites are few, and the amount of data is relatively insufficient. It is difficult to accurately identify pollution characteristics in a small area, which brings certain inconvenience to environmental management. Therefore, analyzing large-scale air data collected by dense microsites will become a promising or even mainstream methodology soon. However, we believe that micro-monitoring sites should not replace state-controlled monitoring sites. After all, state-controlled monitoring sites are mainly aimed at construction and operations projects that involve large investments, large pollutant emissions, serious pollutant hazards, and are likely to cause major environmental harm projects. Instead, the micro-monitoring station is an effective supplement and support for state-controlled monitoring sites. Only with the micro-monitoring station can our environment be accurately protected. The deployment of micro-monitoring sites can further improve the response speed and executive level of the Environmental Protection Agency. Therefore, these two monitoring methods can complement each other and benefit each other.

In this paper, we abstract the O_3_ pollution in Qilihe District, Lanzhou City into a complex network based on the data of 63 micro-monitoring sites in the area. The purpose of this is to more accurately excavate the O_3_ interaction mechanism in the area. With the support of data, the mining of pollution characteristics in the O_3_ network becomes easier and more accurate. The top 10 nodes sorted by node importance are mainly distributed in Agan Town, Xiguoyuan Town, and Bali Town in Qilihe District. These key nodes can help us quickly locate areas with severe pollution and strong transmission capabilities. The above-mentioned node belongs to the area located in the southeast of Qilihe District, Lanzhou City, and all belong to adjacent areas geographically. The correlation between the regions is strong, and the degree of diffusion and transportation of pollutants between regions is also strong. Therefore, cutting off the path between two key nodes can effectively prevent the transmission of pollutants in different regions and control pollution in a certain range. By analyzing the most relevant sub-network in the network, we believe that the degree of mutual influence of pollutants in a small area is not the same. These areas cannot be treated as a whole and cannot receive the same control measures for O_3_ pollution. Therefore, environmental managers should take targeted measures against the region, avoiding the extensive and blind governance based on administrative planning in the past.

In addition, the results of this study are more focused on air management. On the one hand, understanding the interaction influence mechanism of pollutants can help environmental managers formulate precise management and control plans for pollutants in the region. According to the specific conditions of each region, formulate appropriate governance methods to break through the traditional blind and extensive governance methods based on administrative divisions. On the other hand, by analyzing the interaction influence mechanism of nodes in the sub-network, we can prioritize the treatment of these critically polluted areas in the network, thereby reducing the transmission efficiency of pollutants.

Yet, our research has certain limitations; this paper is only a preliminary exploration, and many limitations have yet to be resolved. For example, since micro-monitoring sites have only been deployed in recent years, the experimental data in this article is limited. In the next step, we will expand the scope of data and the amount of the data, which will be more beneficial to the construction of the network. In addition, this study only analyzes the interaction of pollutants in the Qilihe District, and the universality of our research results should be tested in other cities.

## Conclusion

This study presents an analysis of the interaction mechanism of O_3_ between monitoring stations for ambient air quality and provides a theoretical basis for the treatment of O_3_ pollution in adjacent areas. The study uses O_3_ mass concentration time series data from Qilihe District, Lanzhou City and builds a spatial correlation network of adjacent areas based on complex network theory. The N_important method and transfer entropy model were used to analyze the interaction of O_3_ between adjacent areas. The following conclusions were reached:

(1) Construction of spatial correlation network. The optimal connection threshold of the network edge was found to be 0.536619. This value was obtained using the complex network theory, the Pearson correlation coefficient, probability density distribution, power-law distribution, and maximum connected subgraph analysis. Based on this threshold, the spatial correlation network of each area in Qilihe District was developed. It was found that most of the nodes have connected edges and individual nodes are in an isolated state. This indicates that these isolated nodes have no influencing relationship with other nodes but have independence in the change of O_3_ mass concentration and cannot be used for O_3_ pollution of these nodes. For joint governance among nodes, a single governance measure should be formulated and adopted. According to the connection of the edges of the spatial correlation network of O_3_ pollution between regions, the area represented by 63 nodes can be divided into three parts. The first part is mainly distributed in the northern urban area of Qilihe District and the nodes represent urban street community-type areas. The activities are frequent and complicated and the monitoring stations are densely distributed. Results from the analysis indicate that O_3_ concentration in the air quality monitoring stations in the region will be affected by various factors. Thus, there will be considerable differences in the degree of O_3_ pollution. Additionally, both the changing pattern of O_3_ concentration and the degree of correlation decreases. The second part is mainly distributed in the mountainous area in the south of the Qilihe District. The nodes represent townships and villages. Human activities are relatively scarce. The monitoring stations are sparsely distributed. The factors affecting the O_3_ concentration of the air quality monitoring stations in the area are few. This results in high similarities in the degree of O_3_ pollution and O_3_ concentration change law. The third part is the area represented by isolated nodes. This part of the area distribution is more dispersed in urban areas and mountain areas. Furthermore, there is no representativeness in this area.

(2) By analyzing the node importance indicators and algorithms such as degree, degree centrality, close centrality, intermediary centrality, and Pagerank algorithm, and because of the deficiency of Pagerank algorithm in calculating the importance of nodes in the network in this paper, Modify it to get the N_important Important method, N_important method can avoid the insufficiency of the above-mentioned node importance indicators and algorithms in this paper, and is more effective in the ordering of node importance. Finally, the top ten most important nodes in the N_important method were used. A threshold value of 0.536619 was used to build a subnet with the strongest correlation and the highest degree of correlation. It was found that the areas represented by these 10 nodes were mainly distributed in the southeast of Qilihe District and were geographically adjacent to each other. The outcome of the study also revealed that the regions are strongly related and the degree of pollutant diffusion and transportation is relatively high.

(3) Based on the fully connected sub-network constructed above, the mutual influence of O_3_ pollution between nodes in the network was analyzed using transfer entropy. Under the conditions with the greatest degree of influence, the most scope of influence area is node 16649, the actual area should be treated as a key target. Site 15741 is most affected by O_3_ pollution in other site areas. Therefore, to control O_3_ pollution in site 15741, there is the need to start with other site areas that affect the site area and eliminate it from the source. Site 15785 was found to be the weakest node affected by the O_3_ pollution of other nodes. Therefore, for node 15785, we must proceed from ourselves and take measures according to the actual situation. Consequently, these areas cannot be treated as a whole and cannot receive the same control measures for O_3_ pollution. Based on the actual conditions of each area, the causes of O_3_ pollution formation in this area should be analyzed and established. Results indicate the need to establish a cooperative governance mechanism between adjacent regions to achieve joint control and governance of regional O_3_ pollution. Furthermore, the implementation of a joint O_3_ pollution early warning mechanism and the establishment of emergency measures to reduce emissions of O_3_ prerequisites (NO_x_ and VOCs) is crucial for improving air quality in the region.

## Supplemental Information

10.7717/peerj.12095/supp-1 Supplemental Information 1 Raw data. O3 data from the air monitoring station used by the study over a period of one year.

## Additional Information and Declarations

### Data Availability

The following information was supplied regarding data availability: The raw data is available as a Supplemental File.
